# Development and validation of a novel clinical-radiological-pathological scoring system for preoperative prediction of extraprostatic extension in prostate cancer: a multicenter retrospective study

**DOI:** 10.1186/s40644-025-00905-w

**Published:** 2025-07-01

**Authors:** Liqin Yang, Pengfei Jin, Ximing Wang, Zhiping Li, Huijing Xu, Yongsheng Zhang, Feng Cui

**Affiliations:** 1 Department of Radiology, Hangzhou Traditional Chinese Medicine Hospital Affiliated to Zhejiang Chinese Medical University, 453# Tiyuchang Road, Hangzhou, 310007 China; 2 Department of Radiology, Zhejiang Cancer Hospital, Hangzhou Institute of Medicine (HIM), Chinese Academy of Sciences, Hangzhou, 310022 China; 3 Department of Radiology, The First Affiliated Hospital of Soochow University, Suzhou, Jiangsu 215006 China

**Keywords:** Prostate Cancer, Extraprostatic Extension, Scoring System, Risk Stratification

## Abstract

**Objective:**

To develop and validate a multimodal scoring system integrating clinical, radiological, and pathological variables to preoperatively predict extraprostatic extension (EPE) in prostate cancer (PCa).

**Methods:**

This retrospective study included 667 PCa patients divided into a derivation cohort and two validation cohorts. Evaluated parameters comprised prostate-specific antigen density (PSAD), curvilinear contact length (CCL), lesion longest diameter (LD), National Cancer Institute EPE grade (NCI_EPE), International Society of Urological Pathology grade (ISUP), and other relevant variables. Independent predictors were identified through univariate and multivariate regression analysis to construct a logistic model. Coefficients from this model were then weighted to establish a scoring system. The predictive performance of the NCI_EPE, logistic model, and scoring system was systematically evaluated and compared. Finally, the scoring system was stratified into four distinct risk categories.

**Results:**

Multivariate analysis identified NCI_EPE, PSAD, CCL/LD, and ISUP as independent predictors of EPE. In the derivation and validation cohorts, the scoring system demonstrated robust predictive accuracy for EPE, with AUCs of 0.849, 0.830, and 0.847, respectively. These values outperformed the NCI_EPE (Derivation cohort: 0.849 vs. 0.750, *P* < 0.003, Validation cohort 1: 0.830 vs. 0.736, *P* = 0.138, Validation cohort 2: 0.837 vs. 0.715, *P* = 0.003) and were comparable to the logistic model (Derivation cohort: 0.849 vs. 0.860, *P* = 0.228, Validation cohort 1: 0.830 vs. 0.849, *P* = 0.711, Validation cohort 2: 0.837 vs. 0.843, *P* = 0.738). Decision curve analysis revealed higher net clinical benefit for both the scoring system and logistic model compared to the NCI_EPE. Risk stratification using the scoring system categorized patients into four tiers: low (0–3), intermediate-low (4–6), intermediate-high (7–9), and high risk (10–12) with corresponding mean EPE probabilities of 9.9%, 26.0%, 52.0%, and 85.0%. These probabilities closely aligned with observed pT3 incidences in the derivation and validation cohorts.

**Conclusions:**

The scoring system provides enhanced predictive accuracy for EPE, preoperatively stratifying patients into distinct risk categories to facilitate personalized therapeutic strategies.

**Supplementary Information:**

The online version contains supplementary material available at 10.1186/s40644-025-00905-w.

## Introduction

Prostate cancer (PCa) ranks as the most commonly diagnosed malignancy in males across over half of all countries globally. In 2022 alone, it accounted for approximately 1.5 million new cases, with incidence rates continuing to rise annually [1]. The invasiveness of PCa directly influences clinical decision-making, including disease staging and surgical planning, which collectively determine patient prognosis. Extraprostatic extension (EPE), a pathological hallmark of locally advanced PCa, is strongly associated with adverse oncological outcomes, including positive surgical margins (PSM), biochemical recurrence (BCR), and elevated cancer-specific mortality [2]. Nerve-sparing radical prostatectomy (NSRP) offers a greater chance of retaining potency and urinary continence. However, NSRP is contraindicated in patients with PCa suspected of EPE positive, as it may elevate the risk of PSM or BCR, thereby adversely impacting prognosis [3]. Consequently, developing noninvasive and reliable preoperative tools to predict EPE is imperative for balancing oncological safety with functional preservation, enabling tailored surgical planning.

Previous studies have established prediction models for EPE, such as the Partin Scale, Cancer of the Prostate Risk Assessment (CAPRA) score, and Memorial Sloan Kettering Cancer Center (MSKCC) nomogram, primarily based on clinical variables [4–6]. However, these models demonstrated moderate predictive accuracy (AUC: 0.609–0.805) and inconsistent correlation with final pathological outcomes, limiting their clinical utility [7]. Magnetic Resonance Imaging (MRI) plays a pivotal role in PCa diagnosis and has shown superior performance over clinically derived models for predicting EPE [8, 9]. The National Cancer Institute EPE grade (NCI_EPE) proposed by Mehralivand et al. was introduced to improve MRI-based EPE detection, employing standardized quantitative imaging parameters [10]. Despite these advancements, the predictive accuracy of subjective MRI interpretation remains constrained by variability in image quality, heterogeneous EPE imaging features, and inter-observer discrepancies in radiological expertise [11, 12]. Despite these efforts, studies combining radiological or radiomics features with clinical parameters to improve EPE prediction accuracy have predominantly employed single-center validation, and their complexity limits clinical adoption [13–16]. To address these limitations, this multicenter study aims to develop and validate a clinically practical scoring system integrating clinical, radiological, and pathological variables for preoperative predicting EPE.

## Materials and methods

### Study population

This retrospective study, approved by the Institutional Ethics Committee in accordance with the Declaration of Helsinki, waived the requirement for written informed consent.

We enrolled 1459 patients with pathological confirmed PCa who underwent radical prostatectomy (RP) between January 2021 and December 2024 across three institutions (Institution 1: *n* = 670, Institution 2: *n* = 329, Institution 3: *n* = 460). Exclusion criteria were: (1) Incomplete pathological reports precluding definitive EPE assessment (Institution 1: *n* = 126, Institution 2: *n* = 98, Institution 3: *n* = 132); (2) History of prior interventions (e.g., biopsy, surgery, or androgen deprivation therapy) preceding MRI (Institution 1: *n* = 55, Institution 2: *n* = 31, Institution 3: *n* = 48); (3) Poor-quality MRI images (e.g., susceptibility artifacts, motion degradation) compromising radiological interpretation (Institution 1: *n* = 88, Institution 2: *n* = 94, Institution 3: *n* = 120).

Finally, a total of 401 PCa patients from Institution 1 were designated as the derivation cohort for the development of scoring system. External validation was performed using two independent cohorts: validation cohort 1 (*n* = 106, Institution 2) and validation cohort 2 (*n* = 160, Institution 3), to assess the robustness and generalizability of the scoring system.

### Clinical and pathological variables

Clinical variables [age and the preoperative prostate-specific antigen (PSA)] were independently abstracted from medical records by researchers blinded to imaging data. Prostate-specific antigen density (PSAD) was calculated as serum PSA divided by prostate volume (PV). Consistent with established evidence demonstrating MRI's superior correlation with surgical specimen volumes and transrectal ultrasound (TRUS)'s tendency to underestimate PV [17], we utilized MRI-derived volumetry for all PV measurements.

Pathological assessment utilized the International Society of Urological Pathology (ISUP) grades from preoperative biopsies. All participants underwent standardized 10–12 core TRUS-guided systematic biopsies (SB) performed by an interventional ultrasonographer (5–8 years' experience). For patients with PI-RADS ≥ 3 lesions, supplementary TRUS-MRI fusion or cognitive fusion-targeted biopsies of index lesions were obtained. All specimens were processed according to ISUP standardized guidelines.

### MRI acquisition protocol

All institutions performed 3.0 T MRI using phased-array body coils (Institution 1: Philips Achieva; Institution 2: Philips Ingenia; Institution 3: GE Healthcare, Discovery 750 W), adhering to Prostate Imaging Reporting and Data System version 2.1 (PI-RADS v2.1) protocols [18]. Patients received 20 mg of intravenous hyoscine butylbromide (Buscopan®) 1–2 min prior to scanning to suppress bowel peristalsis and they were instructed to empty their bladder naturally 1 h before the examination. Standardized sequences included T2-weighted imaging (T2WI), high b-value diffusion-weighted imaging (DWI) with apparent diffusion coefficient (ADC) maps, and axial dynamic contrast-enhanced (DCE) imaging. For DCE, a Gd-based contrast agent was administered intravenously at 0.1 mmol/kg body weight, with an injection rate of 2–3 mL/s and temporal resolution of 10–15 s. MRI sequence parameters are detailed in Supplementary Table S1.

### Image analysis

Two experienced radiologists [Reader 1 (L.Q.Y.) and Reader 2 (P.F.J.): 3 and 5 years of prostate MRI experience, respectively] independently assessed PI-RADS v2.1 scores and NCI_EPE grade [10, 18], blinded to pathological results. Discordant interpretations were resolved by a third senior radiologist (10 years of prostate MRI expertise, X.M.W), with final determinations reached through consensus. All radiologists were underwent joint training using NCI_EPE, PI-RADS, and lesion measurements before the assessment.

All measurements about lesions were obtained based on the index lesion. Evidence suggested secondary lesion-targeted biopsies provide limited additional diagnostic value when index lesion-targeted biopsies and SB had already performed [19]. Although PCa exhibits multifocality, the index lesion is the core factor driving disease progression. In addition, clinical treatment decisions (e.g., surgical planning) also prioritize the index lesion as the driver of adverse pathology. The study therefore focused the analysis exclusively on index lesions, excluding secondary lesions. To standardize identification, the index lesion was defined as the dominant nodule with either the highest PI-RADS score or largest diameter.

We obtained the following parameters from MRI images: PV, calculated via the ellipsoid formula (π/6 × anteroposterior × transverse × longitudinal diameters on T2WI images); location, classified as peripheral zone (PZ), transition zone (TZ), or PZ + TZ; longest diameter (LD); shortest diameter (SD); lesion volume (LV), manually segmented using ITK-SNAP (v3.4.0; www.itk-snap.org); ADC_mean, quantified from ADC maps; capsular contact length (CCL), defined as the maximal curvilinear tumor-capsule interface, measured using Bessel curve reconstruction on axial T2WI.

The NCI_EPE stratify extracapsular involvement into four grades based on MRI findings [10]: Grade 0: no capsular contact or CCL < 15 mm; Grade 1: CCL ≥ 15 mm or capsular irregularity/bulge; Grade 2: CCL ≥ 15 mm and capsular irregularity/bulge; Grade 3: Unequivocal evidence of capsular disruption with tumor extension into periprostatic fat or direct invasion of adjacent structures.

Categorical variables in the final analysis were based on consensus interpretations, while continuous variables were represented as mean values derived from both readers’ measurements to mitigate inter-reader variability.

### Radiological-pathological correlation

Pathological evaluation of RP specimens by a dedicated genitourinary pathologist served as the reference standard for EPE diagnosis. EPE status was extracted from surgical pathology reports. To ensure precise spatial correlation between MRI-identified lesions and pathology, a senior urologist experienced in PCa diagnosis supervised the alignment of radiological and pathological findings. Only MRI-visible lesions with confirmed pathological correspondence were retained for analysis. EPE was pathologically defined as tumor extension beyond the prostate capsule, which was considered as pT3. Conversely, those without EPE were pT2.

### Statistical analysis

Analyses were performed using R software (v4.1.0). Continuous variables were expressed as mean ± standard deviation or median (interquartile range [IQR]), with comparisons made via independent T-tests or Mann–Whitney U tests, contingent on normality assessed by the Shapiro–Wilk test. Categorical variables were reported as frequencies (percentages) and analyzed using chi-square tests.

In the derivation cohort, variables with statistically significance between pT2 and pT3 were dichotomized using receiver operating characteristic (ROC)-derived optimal thresholds or clinical consensus. These variables were retained for univariate and multivariate logistic regression analyses after confirming no multicollinearity. Independent predictors identified through this analysis were used to construct the logistic model, and their regression coefficients (β) were subsequently weighted to establish the scoring system: each variable’s score = β/β_min_ × 2, where β_min_ represents the smallest coefficient among retained variables [20, 21]. Total scores were calculated by summing individual weighted scores.

The area under the curve (AUC), sensitivity, specificity, positive predictive value (PPV), negative predictive value (NPV), and accuracy of the NCI_EPE grade, logistic model, and scoring system were calculated. Comparisons were conducted using the DeLong test for AUC differences and the *P* values of multiple testing among the three groups were adjusted using the Bonferroni correction. Hosmer–Lemeshow test was employed to assess the agreement between the scoring system and pathological results. Decision curve analysis (DCA) evaluated net clinical benefit across probability thresholds. *P* < 0.05 considered statistically significant.

## Results

### Baseline characteristics

This study enrolled 667 patients with PCa, stratified into a derivation cohort (*n* = 401) and two validation cohorts (*n* = 106 and *n* = 160). pT3 incidences were comparable across cohorts: 41.4% (166/401) in the derivation cohort, 41.5% (44/106) in validation cohort 1, and 41.3% (66/160) in validation cohort 2. No significant intercohort differences were observed in age, ADC_mean, PSA, PV, PSAD, position, LV, LD, SD, CCL, CCL/LD, NCI_EPE, PI-RADS, or ISUP (all *P* > 0.05, Table 1).

**Table 1:** The general data of patients in the derivation and validation cohorts

Characteristics	Derivation cohort	Validation cohort 1	Validation cohort 2	*P* value
n	401	106	160	
label, n (%)				0.999
pT2	235 (58.6%)	62 (58.5%)	94 (58.8%)	
pT3	166 (41.4%)	44 (41.5%)	66 (41.3%)	
Age (year), median (IQR)	71 (66, 74)	70.5 (66, 74)	71 (65, 75)	0.943
ADC_Mean, median (IQR)	0.86 (0.68, 1.03)	0.81 (0.62, 1.06)	0.88 (0.72, 1.06)	0.427
Position, n (%)				0.727
PZ	212 (52.9%)	52 (49.1%)	82 (51.3%)	
TZ	129 (32.2%)	41 (38.7%)	52 (32.5%)	
PZ + TZ	60 (15.0%)	13 (12.3%)	26 (16.3%)	
SD (mm), median (IQR)	10.40 (7.70, 14.60)	10.75 (8.20, 16.63)	11.15 (7.90, 15.40)	0.531
NCI_EPE, n (%)				0.060
0	107 (26.7%)	25 (23.6%)	48 (30.0%)	
1	99 (24.7%)	22 (20.8%)	20 (12.5%)	
2	149 (37.2%)	48 (45.3%)	69 (43.1%)	
3	46 (11.5%)	11 (10.4%)	23 (14.4%)	
PSA (ng/ml), median (IQR)	13.88 (8.62, 26.30)	13.70 (8.15, 30.43)	14.40 (9.31, 26.40)	0.927
PV (ml), median (IQR)	38.47 (28.45, 54.49)	35.81 (26.49, 53.95)	38.80 (27.71, 58.85)	0.496
PSAD (ng/ml/cm^3^), median (IQR)	0.35 (0.20, 0.78)	0.375 (0.20, 0.805)	0.335 (0.21, 0.81)	0.948
CCL (mm), median (IQR)	21.80 (9.80, 36.70)	24.65 (12.45, 34.80)	23.50 (9.48, 29.43)	0.188
LD (mm), median (IQR)	17.50 (12.40, 25.80)	18.65 (14.73, 26.03)	17.50 (12.00, 27.33)	0.410
CCL/LD, median (IQR)	1.23 (0.70, 1.69)	1.26 (0.91, 1.67)	1.10 (0.64, 1.57)	0.106
LV (ml), median (IQR)	1.53 (0.66, 4.45)	1.78 (0.85, 4.30)	1.58 (0.71, 4.61)	0.509
PI-RADS, n (%)				0.669
2	49 (12.2%)	14 (13.2%)	23 (14.4%)	
3	34 (8.5%)	9 (8.5%)	13 (8.1%)	
4	93 (23.2%)	16 (15.1%)	31 (19.4%)	
5	225 (56.1%)	67 (63.2%)	93 (58.1%)	
ISUP, n (%)				0.596
1	37 (9.2%)	10 (9.4%)	19 (11.9%)	
2	93 (23.2%)	32 (30.2%)	43 (26.9%)	
3	108 (26.9%)	26 (24.5%)	48 (30.0%)	
4	79 (19.7%)	17 (16.0%)	23 (14.4%)	
5	84 (20.9%)	21 (19.8%)	27 (16.9%)	

*PSA* Prostate specific antigen, *PV* Prostate volume, *PSAD* Prostate specific antigen density, *PZ* Peripheral zone, *TZ* Transition zone, *LV* Lesion volume, *LD* Longest diameter, *SD* Shortest diameter, *CCL* Curvilinear contact length, *NCI_EPE* National Cancer Institute extraprostatic extension, *PI-RADS* Prostate Imaging Report and Data System version, *ADC* Apparent Diffusion Coefficient, *ISUP* International Society of Urological Pathology

### Development of the scoring system

The inter-group analysis in the derivation cohort (Table 2) showed that there were statistically significant differences between pT2 and pT3 in terms of position, SD, LD, CCL, CCL/LD, PSA, PSAD, LV, NCI_EPE, PI-RADS, and ISUP (*P* < 0.05). Among them, PSA, CCL and LD were excluded in the subsequent analyses due to the multicollinearity with PSAD and CCL/LD. Then, the retained variables were dichotomized via ROC curve analysis (Supplementary Table S2). PSAD was categorized using a clinically cutoff (≥ 0.20 ng/mL/cm^3^).

**Table 2:** The general data of patients in the derivation cohort

Characteristics	pT2	pT3	*P* value
n	235 (58.6%)	166 (41.4%)	
Age (year), median (IQR)	71 (66, 74)	71 (66, 75)	0.622
ADC_Mean, median (IQR)	0.85 (0.65, 1.07)	0.87 (0.71, 1.01)	0.793
Position, n (%)			< 0.001
PZ	125 (53.2%)	87 (52.4%)	
TZ	96 (40.9%)	33 (19.9%)	
PZ + TZ	14 (6.0%)	46 (27.7%)	
SD (mm), median (IQR)	9.1 (7.0, 12.7)	12.35 (9.15, 20.5)	< 0.001
NCI_EPE, n (%)			< 0.001
0	93 (39.6%)	14 (8.4%)	
1	63 (26.8%)	36 (21.7%)	
2	75 (31.9%)	74 (44.6%)	
3	4 (1.7%)	42 (25.3%)	
PSA (ng/ml), median (IQR)	10.75 (7.43, 17.95)	22.85 (12.01, 42.00)	< 0.001
PV (ml), median (IQR)	39.35 (28.97, 56.80)	36.43 (26.92, 50.72)	0.051
PSAD (ng/ml/cm^3^), median (IQR)	0.26 (0.17, 0.44)	0.66 (0.34, 1.26)	< 0.001
CCL (mm), median (IQR)	16.20 (5.35, 26.20)	33.65 (19.50, 57.96)	< 0.001
LD (mm), median (IQR)	14.7 (10.95, 20.65)	23.30 (16.13, 32.50)	< 0.001
CCL/LD, median (IQR)	1.08 (0.36, 1.42)	1.53 (1.09, 1.93)	< 0.001
LV (ml), median (IQR)	1.04 (0.44, 2.91)	3.06 (1.11, 7.82)	< 0.001
PI-RADS, n (%)			< 0.001
2	45 (19.1%)	4 (2.4%)	
3	29 (12.3%)	5 (3.0%)	
4	58 (24.7%)	35 (21.1%)	
5	103 (43.8%)	122 (73.5%)	
ISUP, n (%)			< 0.001
1	32 (13.6%)	5 (3.0%)	
2	75 (31.9%)	18 (10.8%)	
3	77 (32.8%)	31 (18.7%)	
4	27 (11.5%)	52 (31.3%)	
5	24 (10.2%)	60 (36.1%)	

*PSA* Prostate specific antigen, *PV* Prostate volume, *PSAD* Prostate specific antigen density, *PZ* Peripheral zone, *TZ* Transition zone, *LV* Lesion volume, *LD* Longest diameter, *SD* Shortest diameter, *CCL* Curvilinear contact length, *NCI_EPE* National Cancer Institute extraprostatic extension, *PI-RADS* Prostate Imaging Report and Data System version, *ADC* Apparent Diffusion Coefficient, *ISUP* International Society of Urological Pathology

Univariate and multivariate logistic regression analyses (Table 3) identified NCI_EPE (≥ 2), PSAD (≥ 0.20), CCL/LD (> 1.34), and ISUP (≥ 4) as independent predictors of EPE, forming the logistic model. Each independent predictor was assigned a weighted score using the formula: ß/ß_min_ × 2. Scores were allocated as follows: NCI_EPE (≥ 2) = 2 (ß = 1.031), PSAD (≥ 0.20) = 3 (ß = 1.261), CCL/LD (> 1.34) = 2 (ß = 0.946), and ISUP (≥ 4) = 5 (ß = 2.183). The total score of the scoring system ranged from 0 to 12, with incremental values reflecting escalating EPE risk.

**Table 3:** The univariate and multivariate analyses in the derivation cohort and weighted points

Characteristics	Total (N)	Univariate analysis		Multivariate analysis			
		Odds Ratio (95% CI)	*P* value	ß	Odds Ratio (95% CI)	*P* value	Weighted score
NCI_EPE	401						
< 2	206	Reference			Reference		
≥ 2	195	4.581 (2.986–7.029)	** < 0.001**	1.031	2.803 (1.512–5.195)	**0.001**	2
LV (ml)	401						
< 1.89	240	Reference			Reference		
≥ 1.89	161	2.735 (1.809–4.135)	**< 0.001**		0.683 (0.375–1.245)	0.213	
PSAD (ng/ml/cm^3^)	401						
< 0.20	91	Reference			Reference		
≥ 0.20	310	4.028 (2.273–7.140)	**< 0.001**	1.261	3.528 (1.759–7.076)	**< 0.001**	3
CCL/LD	401						
< 1.34	227	Reference			Reference		
≥ 1.34	174	4.547 (2.971–6.961)	**< 0.001**	0.946	2.576 (1.472–4.509)	**< 0.001**	2
PI-RADS	401						
< 5	176	Reference			Reference		
≥ 5	225	3.553 (2.311–5.464)	** < 0.001**		1.563 (0.846–2.890)	0.154	
SD (mm)	401						
< 8.95	152	Reference			Reference		
≥ 8.95	249	3.341 (2.139–5.220)	** < 0.001**		1.498 (0.805–2.790)	0.202	
ISUP	401						
< 4	238	Reference			Reference		
≥ 4	163	7.483 (4.776–11.724)	**< 0.001**	2.183	8.870 (5.160–15.247)	**< 0.001**	5

*NCI_EPE* National Cancer Institute extraprostatic extension, *LV* Lesion volume, *PSAD* Prostate specific antigen density, *CCL* Curvilinear contact length, *LD* Longest diameter, *PI-RADS* Prostate Imaging Report and Data System version, *SD* Shortest diameter, *ISUP* International Society of Urological Pathology, *CI* Confidence interval, *ß* correlation coefficient. Data in bold indicates *P* < 0.05

### Predictive performance comparison

**Fig. 1:**
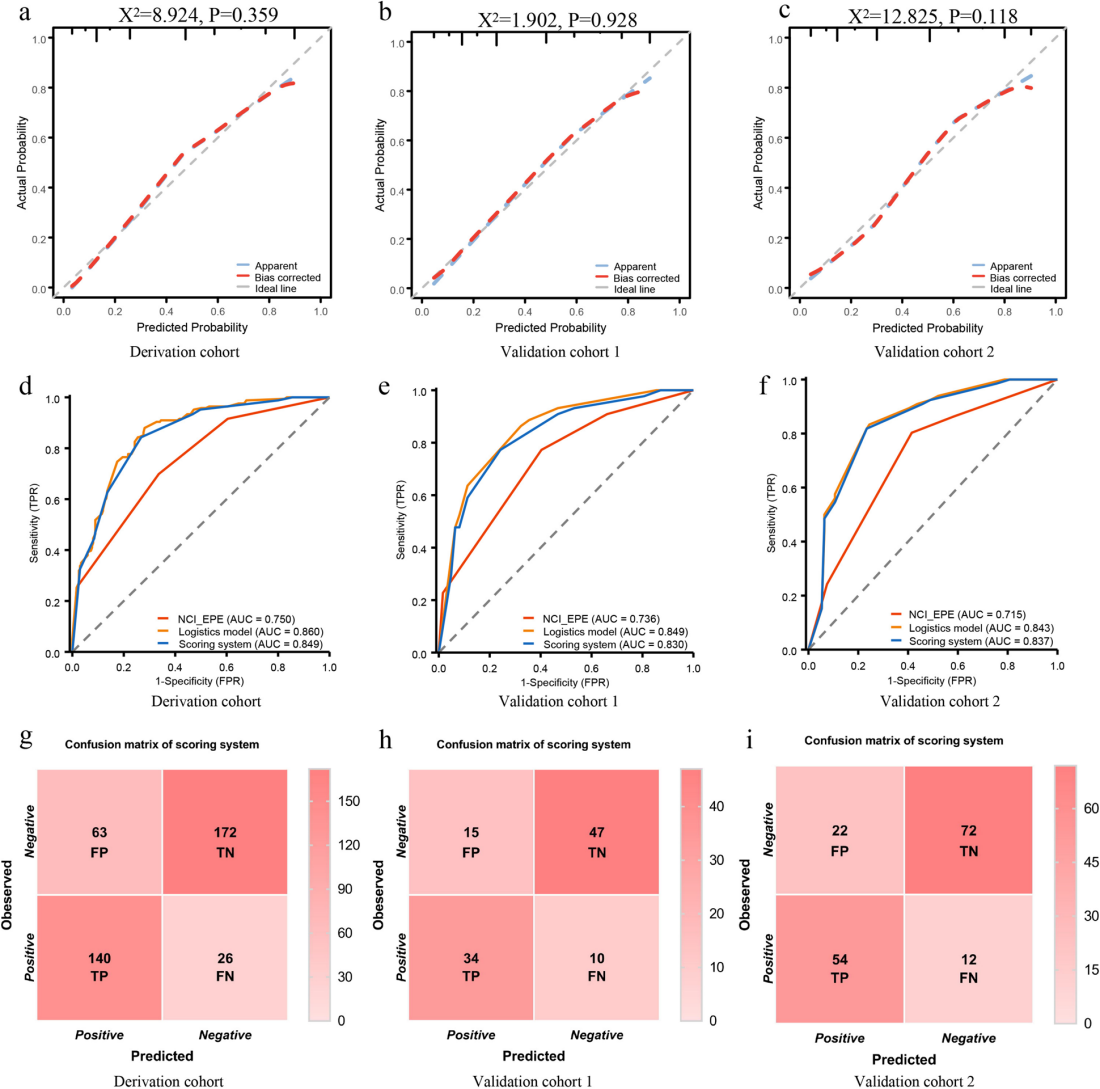
**a**-**c** The calibration curve for the evaluation of EPE between the scoring system and the pathological results in the derivation cohort, validation cohort 1 and validation cohort 2. **d**-**f** The comparison of the performance among NCI_EPE, logistic model and scoring system in the derivation cohort, validation cohort 1 and validation cohort 2. **g**-**i** The confusion matrix distribution diagram of the prediction of EPE by scoring system in the derivation cohort, validation cohort 1 and validation cohort 2. EPE: Extraprostatic extension, NCI_EPE: National Cancer Institute extraprostatic extension, AUC: areas under the curve,TP: true positive, TN: true negative, FP: false positive, FN: false negative

The Hosmer–Lemeshow test demonstrated good calibration of the scoring system with pathological EPE outcomes across all cohorts (*P* = 0.348 [derivation], 0.928 [validation 1], and 0.118 [validation 2]; Fig. 1: a-c). Optimal thresholds for discriminating EPE risk were consistent across cohorts: ≥ 2 for the NCI_EPE, ≥ 0.36 probability for the logistic model, and ≥ 6 points for the scoring system.

The NCI_EPE demonstrated suboptimal performance across all cohorts, with AUC values ranging only from 0.715 to 0.750. In the derivation cohort, specificity and PPV for NCI_EPE were low (specificity of 0.663 and PPV of 0.595). However, both the logistic model and scoring system significantly improved these metrics (specificity: 0.719 and 0.732; PPV: 0.689 and 0.690, respectively). Similar trends were observed in the validation cohorts, where the NCI_EPE exhibited limited specificity and PPV, while the logistic model and scoring system achieved marked enhancements. The logistic model and scoring system showed comparable predictive performance (Derivation cohort: 0.860 vs. 0.849, *P* = 0.228, Validation cohort 1: 0.849 vs. 0.830, *P* = 0.711, Validation cohort 2: 0.843 vs. 0.837, *P* = 0.738), significantly outperforming the NCI_EPE (Derivation cohort: 0.860 and 0.849 vs. 0.750, both *P* < 0.003, Validation cohort 1: 0.849 and 0.830 vs. 0.736, *P* = 0.024 and 0.138, Validation cohort 2: 0.843 and 0.837 vs. 0.715, *P* < 0.003 and *P* = 0.003). Comprehensive results were illustrated in Fig. 1, d–f and summarized in Table 4.

**Table 4:** The comparison of the performance among NCI_EPE grade, logistic model and scoring system in the derivation and validation cohorts

**Groups**	Derivation cohort			Validation cohort 1			Validation cohort 2		
**Schemes**	NCI_EPE	Logistic model	Scoring system	NCI_EPE	Logistic model	Scoring system	NCI_EPE	Logistic model	Scoring system
cut-off	≥ 2	≥ 0.36	≥ 6	≥ 2	≥ 0.36	≥ 6	≥ 2	≥ 0.36	≥ 6
Sensitivity	0.699	0.880	0.843	0.773	0.864	0.773	0.803	0.833	0.818
Specificity	0.663	0.719	0.732	0.597	0.677	0.758	0.585	0.755	0.766
Accuracy	0.678	0.786	0.778	0.67	0.755	0.764	0.675	0.788	0.788
PPV	0.595	0.689	0.690	0.576	0.655	0.694	0.576	0.705	0.711
NPV	0.757	0.894	0.869	0.787	0.875	0.825	0.809	0.866	0.857
AUC (95% CI)	0.750 (0.704- 0.795)	0.860 (0.824- 0.896)	0.849 (0.812- 0.886)	0.736 (0.647- 0.825)	0.849 (0.777- 0.921)	0.830 (0.752- 0.908)	0.715 (0.640- 0.791)	0.843 (0.782- 0.904)	0.837 (0.774- 0.899)
*P* value^*^	** < 0.003**^**a**^	0.228^b^	** < 0.003**^**c**^	**0.024**^**a**^	0.711^b^	0.138^c^	** < 0.003**^**a**^	0.738^b^	**0.003**^**c**^

Data in bold indicates *P* < 0.05

*NCI_EPE* National Cancer Institute extraprostatic extension, *AUC* Areas under the curve, *CI* Confidence interval, *PPV* Positive predictive value, *NPV* Negative predictive value

^*^the *P* values were adjusted using the Bonferroni correction

^a^The differences in AUC between NCI_EPE grade and logistic model

^b^The differences in AUC between logistic model and Scoring system

^c^The differences in AUC between NCI_EPE grade and Scoring system

**Fig. 2:**
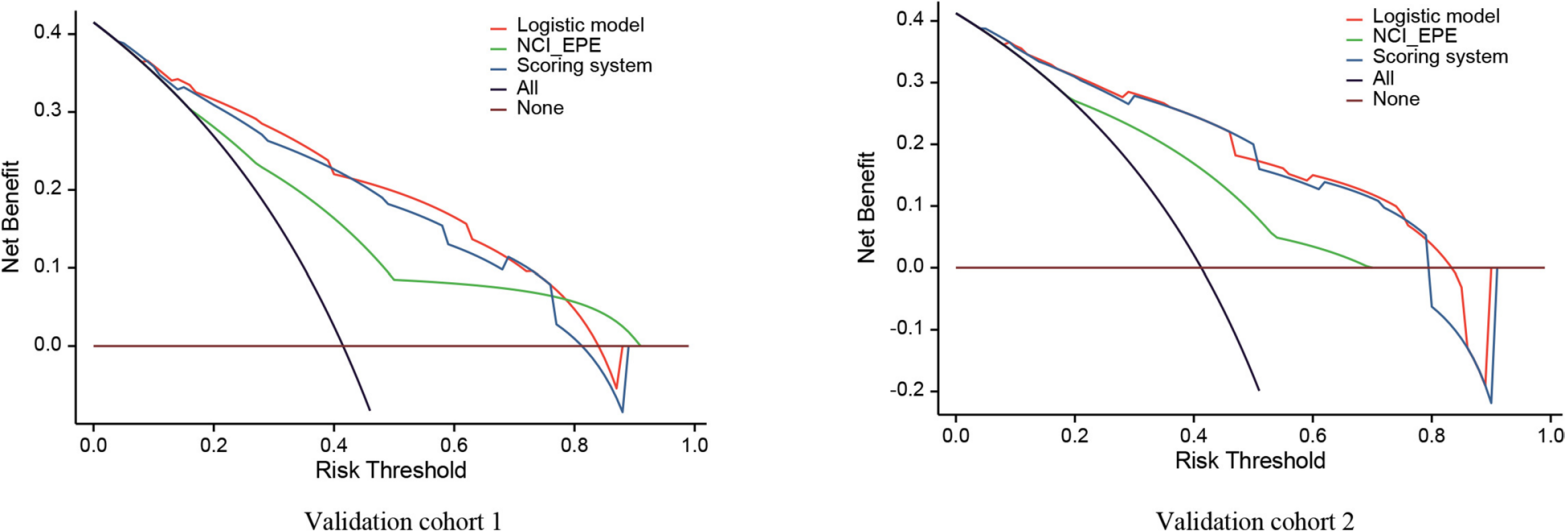
The decision curve analysis among the NCI_EPE, logistic model and scoring system in the validation cohort 1 and validation cohort 2. NCI_EPE: National Cancer Institute extraprostatic extension

The scoring system achieved recall rates of 0.843 (140/166), 0.773 (34/44), and 0.818 (54/66) in the derivation cohort, validation cohort 1, and validation cohort 2, respectively. Precision were 0.690 (140/203), 0.694 (34/49), and 0.711 (54/76) across these cohorts, indicating a minimal likelihood of false-negative results (Fig. 1, g-i). DCA further demonstrated that the scoring system provided net clinical benefits equivalent to the logistic model in both validation cohorts, with both tools outperforming the NCI_EPE across a wide probability threshold range (Fig. 2).

### Risk stratification

The scoring system stratified patients into four risk tiers based on total scores, corresponding to escalating incidences of pT3: low (0–3 points), intermediate-low (4–6 points), intermediate-high (7–9 points), and high risk (10–12 points). The mean prediction probabilities for these tiers were 9.9%, 26.0%, 52.0%, and 85.0%, respectively, closely matching the observed pT3 incidences across all cohorts (Table 5).

**Table 5:** Estimated and observed risks for pT3 according to the scoring system

Risk Group (Total Points)	Total (N)	^*^Estimated risk for pT3	Observed risk for pT3		
			Derivation cohort	Validation cohort 1	Validation cohort 2
Low Risk					
0–3	211	9.9%	6.3% (8/126)	9.4% (3/32)	9.4% (5/53)
Intermediate-low Risk					
4–6	128	26.0%	25.0% (18/72)	28.0% (7/25)	22.6% (7/31)
Intermediate-high Risk					
7–9	172	52.0%	60.6% (66/109)	54.2% (13/24)	59.0% (23/39)
High Risk					
10–12	156	85.0%	78.7% (74/94)	84.0% (21/25)	83.8% (31/37)

*EPE* Extraprostatic extension

^*^Values are the median of the estimated risk for each group

**Fig. 3:**
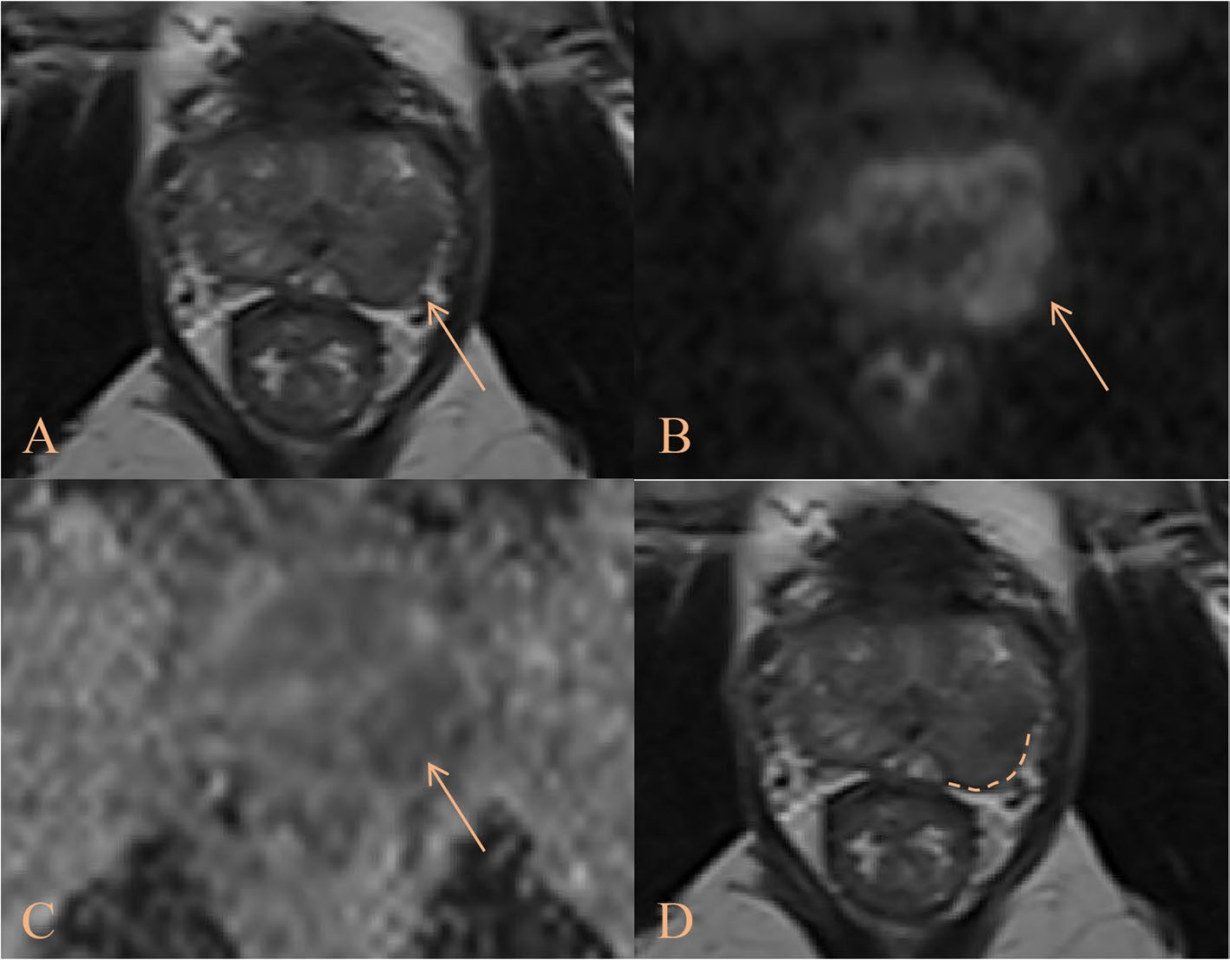
The patient was a 69-year-old male. The A-C images consisted of T2-weighted imaging, diffusion weighted imaging, and apparent diffusion coefficient maps, respectively, with the tumor indicated by the yellow arrow. The D image represented a T2WI image with focal labeling processing, where the curvilinear contact length (CCL) was highlighted by the dashed line. Clinical, radiological and pathological data were as follows: PSA 12.55, PSAD 0.33, CCL 18.5, CCL/LD 1.04, NCI_EPE grade 2, IUSP 2. According to the scoring system, the final value was 5 points, which was divided into intermediate-low tier, and the final pathological result was pT2

**Fig. 4:**
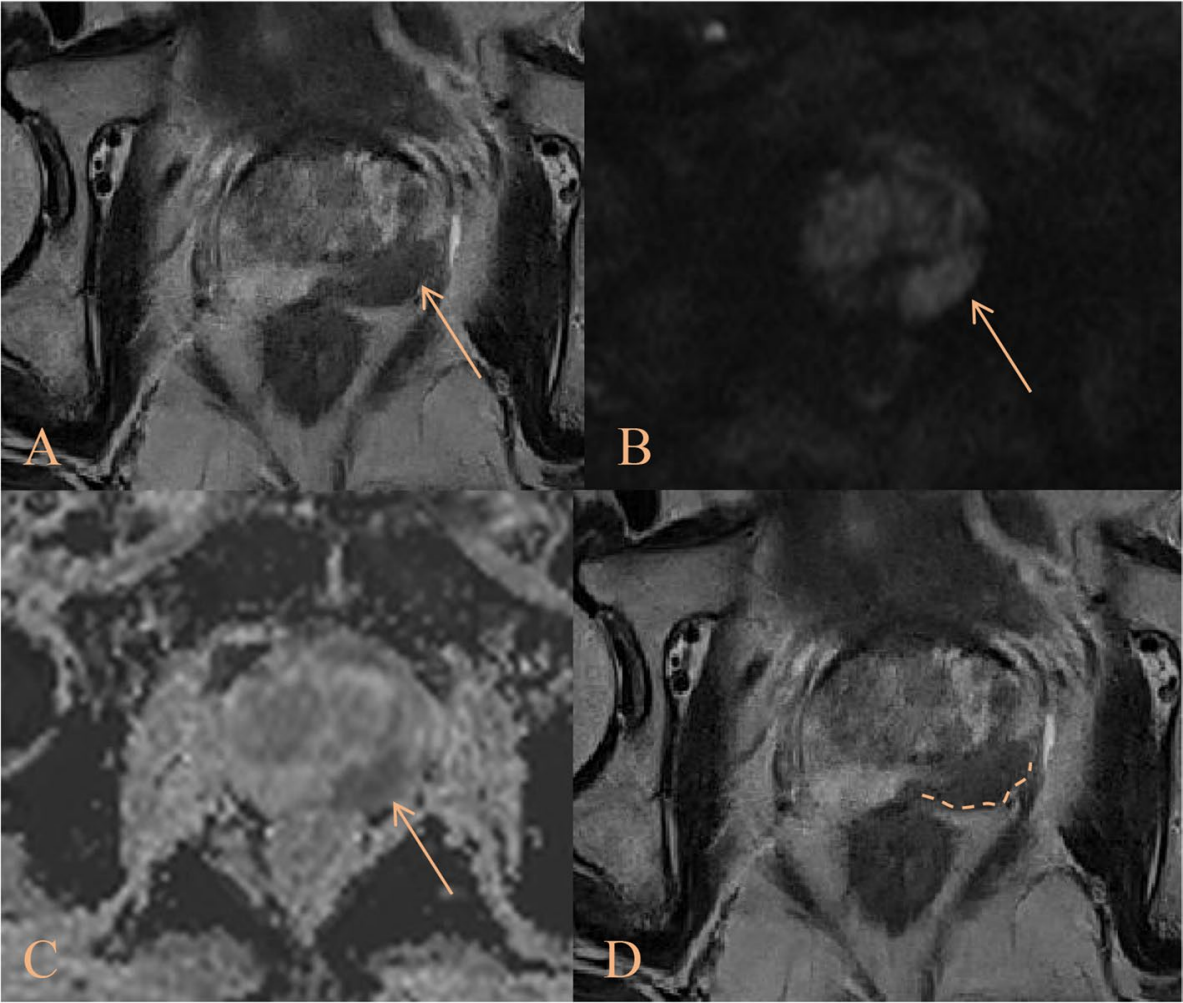
The patient was a 69-year-old male. The A-C images consisted of T2-weighted imaging, diffusion weighted imaging, and apparent diffusion coefficient maps, respectively, with the tumor indicated by the yellow arrow. The D image represented a T2WI image with focal labeling processing, where the curvilinear contact length (CCL) was highlighted by the dashed line. Clinical, radiological and pathological data were as follows: PSA 29.53, PSAD 0.91, CCL 28.2, CCL/LD 1.48, NCI_EPE grade 2, ISUP_GG 4. According to the scoring system, the final value was 7 points, which was divided into intermediate-high tier, and the final pathological result was pT3

Among 266 cases classified as NCI_EPE grade 2 across all cohorts, the scoring system upgraded 181 cases to higher-risk categories, of which 115 were pathologically confirmed as pT3. Conversely, 85 non-upgraded cases included 65 pT2 instances (Supplementary Table S3). This reclassification demonstrated significant risk stratification beyond the NCI_EPE (*P* < 0.001), effectively distinguishing occult high-risk lesions (Figs. 3 and 4).

**Fig. 5:**
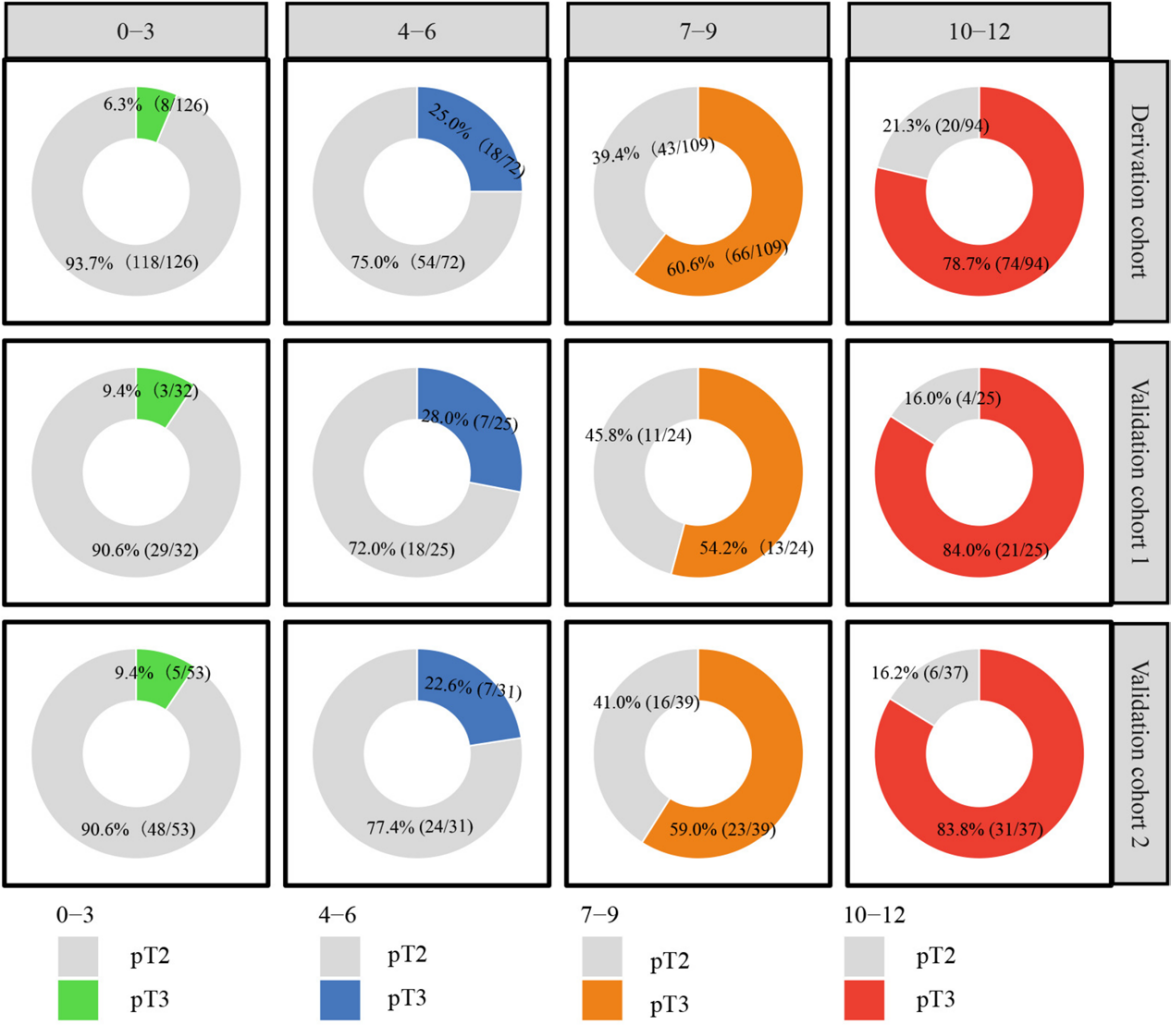
The incidence of EPE in the four risk prediction subgroups for the derivation cohort, validation cohort 1 and validation cohort 2

Based on the risk stratification of scoring system, pT3 incidences across subgroups were shown in Fig. 5. Patients were categorized as low-risk (≤ 6 points) or high-risk (≥ 7 points). For low-risk patients considered for NSRP, undertreatment percentages were 13.1% (26/198), 17.5% (10/57), and 14.3% (12/84) in the derivation cohort and validation cohorts, respectively. Conversely, overtreatment percentages in high-risk patients were 31.0% (63/203), 30.6% (15/49), and 28.9% (22/76).

## Discussion

Accurate preoperative assessment of EPE is critical for evaluating PCa invasiveness and guiding optimal surgical strategies. In this study, we developed a multimodal scoring system integrating four key predictors: NCI_EPE grade, PSAD, CCL/LD, and ISUP grade—collectively capturing clinical, radiological, and pathological characteristics. The scoring system demonstrated superior predictive performance across all cohorts, achieving AUCs of 0.830–0.849 compared to the NCI_EPE (AUC: 0.715–0.750). Notably, it improved specificity and PPV, significantly reducing false-positive rates that could lead to overtreatment. Moreover, the scoring system stratified patients into four distinct risk tiers—low, intermediate-low, intermediate-high, and high—demonstrating robust discriminatory capacity between subgroups and a strong positive correlation between total scores and EPE incidence. This reliable and user-friendly scoring system holds promise for precise clinical diagnosis and treatment for personalized PCa management.

While scoring systems in other fields have been extensively explored [22–25], our study firstly introduced a novel clinical-radiological-pathological scoring system for preoperative prediction of EPE in PCa. Unlike prior models, this scoring system uniquely incorporated the NCI_EPE—a standardized MRI-based grading system for extracapsular involvement—as a foundational element, synergizing it with clinicopathological variables (PSAD, ISUP, and CCL/LD) to enhance preoperative risk stratification. Recently, the NCI_EPE grade has become as a widely recognized tool with significant clinical applicability in predicting EPE [10]. The NCI_EPE grade have demonstrated moderate diagnostic performance in prior studies, with AUC values ranging from 0.70 to 0.85. However, its clinical utility is constrained by the interobserver variability in interpreting subtle MRI features (e.g., capsular irregularity or bulge vs. definitive EPE), and the heterogeneous imaging manifestations of EPE, which often lack clear anatomical demarcation. These factors contribute to inconsistent diagnostic accuracy, particularly in equivocal cases where microscopic invasion eludes radiographic detection [26–28]. The NCI_EPE demonstrated limited predictive accuracy in our cohorts, with AUCs of 0.750 (derivation), 0.736 (validation cohort 1), and 0.715 (validation cohort 2). Despite this, we assigned NCI_EPE grade ≥ 2 a score of 2 points within the multimodal scoring system to retain its contributory value.

The CCL/LD, another radiological variable introduced in this study, refined the established role of CCL as an independent predictor of EPE. While prior reports extensively confirmed the predictive value of CCL in EPE [29, 30], our ratio normalized CCL by LD, effectively controlling for tumor size-related confounding. This standardization accounted for the inherent correlation between larger lesions and increased CCL, reducing overestimation of EPE risk in volumetrically dominant tumors lacking true invasiveness. CCL/LD could enhance the diagnostic specificity, particularly in lesions where absolute CCL may misleadingly suggest EPE. In our scoring system, CCL/LD was ultimately assigned 2 points. Furthermore, analysis of patients with NCI_EPE grade 2 revealed that non-radiological variables (e.g., PSAD, ISUP) significantly enhanced risk stratification, underscoring the necessity of integrating clinical and pathological variables to predict EPE.

PSAD serves as a biomarker of tumor aggressiveness in PCa. Multivariate analysis confirmed PSAD as an independent predictor of EPE (OR = 3.528; *P* < 0.001), with supplementary diagnostic value in MRI-negative or equivocal cases where conventional imaging criteria proved indeterminate. Our cohort-derived PSAD threshold of 0.20 ng/ml/cm^3^ demonstrated optimal performance in high-risk scenarios. This aligned with the previous studies advocating integration of PSAD into multiparametric prediction tools (e.g., MSKCC nomogram) to offset single-marker limitations and improve risk stratification accuracy [6, 31]. Consistent with this rationale, PSAD was assigned 3 points in our scoring system, reflecting its independent association with EPE. PSAD serves as a biomarker of tumor biological activity, whereas the ISUP grade provides critical histopathological stratification of PCa aggressiveness. The ISUP directly correlates with tumor malignancy, strongly linked to pathological hallmarks including stromal destruction, perineural invasion, and EPE. Established evidence indicated EPE incidence approaches 40–60% in patients with ISUP grades 4–5 [32], solidifying its role as a foundational parameter for preoperative risk stratification. Reflecting this prognostic significance, our scoring system assigned ISUP the highest weight (5 points; β = 2.183, *P* < 0.001) among all variables, underscoring its dominant association with EPE risk. Considering these findings, all four independent risk factors ultimately incorporated into our study have been corroborated by various investigations, further affirming the reliability and comprehensiveness of the scoring system.

The proposed scoring system was directly derived from the logistic model. ROC curve analysis confirmed strong concordance between the scoring system and logistic model in EPE prediction, with excellent calibration across all derivation and validation cohorts. Importantly, the scoring system maintains diagnostic accuracy while offering enhanced clinical practicality: physicians could straightforward estimate the probability of EPE by summing variable-specific points from four routinely available parameters (NCI_EPE, PSAD, ISUP, and CCL/LD), avoiding the computational complexity of the logistic model. Furthermore, the scoring system also demonstrated resilience to institutional differences in MRI protocols or patient demographics (AUC: 0.830 and 0.837 [validation cohort 1 and 2] vs 0.849 [derivation cohort], *P* > 0.05), with comparable performance observed in all cohorts. The final risk prediction stratification of the scoring system is also essential. The scoring system categorizes patients into four distinct risk tiers: low risk (0–3 points), intermediate-low risk (4–6), intermediate-high risk (7–9) and high risk (10–12). The mean prediction probabilities for these four tiers were closely matching the observed pT3 incidences across all cohorts. By translating complex regression outputs into ordinal risk categories, this scoring system simplifies preoperative counseling: high-risk patients (≥ 7 points) may warrant wide-margin resection to minimize residual disease, whereas low-risk cases (≤ 6 points) remain eligible for NSRP approaches to preserve functional outcomes. Such stratification standardizes EPE risk communication facilitates accurate pre-operative evaluation of EPE thereby promoting individualized and precise diagnosis and treatment for PCa patients.

Our multimodal scoring system exclusively utilized MRI data, potentially overlooking the complementary value from other imaging modalities. Notably, substantial evidence associated TRUS-detected hypoechoic lesions with the detection of clinically significant PCa (csPCa) [33]. Recent study further indicated that hypoechoic lesions with microcalcifications enhanceed csPCa identification, where microcalcifications correlate with higher-grade disease. Additionally, several studies had shown that cribriform morphology in PCa was predictive of poor prognosis [34]. TRUS outperforms MRI in microcalcification visualization and offers superior accuracy and convenience for 3D volumetric measurements. Given these advantages, TRUS-derived features may improve the EPE prediction. Future research should evaluate whether incorporating TRUS data could improve predictive accuracy.

This study is subject to certain limitations. One aspect, it was a retrospective design, which may introduce inherent selection bias. The uneven distribution of the derivation and validation cohorts across three risk subgroups may potentially introduce bias and limits causal inference, thus it is recommended to develop and validate the scoring system using prospective multicenter validation to confirm generalizability across diverse populations and imaging protocols. On the other hand, it is important to acknowledged that certain scoring factors included in current scoring system rely on the subjective predictions, leading to potential variations among different readers. To address this issue, future researches could explore the integration of AI-driven standardization of imaging parameters (e.g., automated PV measurement, NCI_EPE evaluation or CCL/LD quantification) to minimize observer-dependent variability.

## Conclusion

In summary, this study advanced preoperative EPE risk assessment by integrating clinical, radiological, and pathological variables into a multimodal scoring system. The tool significantly outperformed the NCI_EPE grade while stratifying patients into four distinct risk tiers (low, intermediate-low, intermediate-high, high), enabling personalized treatment decisions through risk-adapted surgical planning.

## Supplementary Information

Supplementary Material 1

## Data Availability

No datasets were generated or analysed during the current study.
