# Supplementary material for: Development and validation of a novel clinical-radiological-pathological scoring system for preoperative prediction of extraprostatic extension in prostate cancer: a multicenter retrospective study
Source: Cancer Imaging. 2025 Jul 1;25:83. doi: 10.1186/s40644-025-00905-w (PMC12220475; doi:10.1186/s40644-025-00905-w)
Supplement: Supplementary file 1 — Supplementary Material 1 [file 40644_2025_905_MOESM1_ESM.docx]

**Supplementaries**

| Table S1 Detailed sequence parameters for prostate multi-parametric MRI in three institutions |
| --- |
| \| Parameter \| Institution 1 \| \| \| Institution 2 \| \| \| Institution 3 \| \| \| \| --- \| --- \| --- \| --- \| --- \| --- \| --- \| --- \| --- \| --- \| \| T2WI \| DWI \| DCE-MRI \| T2WI \| DWI \| DCE-MRI \| T2WI \| DWI \| DCE-MRI \| \| TR (ms) \| 6980 \| 6540 \| 3.22 \| 5639 \| 4000 \| 4.2 \| 11399.8 \| 3417 \| 2.6 \| \| TE (ms) \| 104 \| 64 \| 1,18 \| 125 \| 50 \| 0 \| 99.9 \| 53.7 \| 1.1 \| \| Thickness (mm) \| 3 \| 3 \| 3 \| 3 \| 3 \| 3 \| 3 \| 3 \| 3 \| \| Interslice gap (mm) \| 0 \| 0 \| 0 \| 0 \| 0 \| 0 \| 0 \| 0 \| 0 \| \| Matrix size \| 520×520 \| 169×169 \| 113×113 \| 320×280 \| 184×184 \| 256×256 \| 288×288 \| 128×128 \| 200×160 \| \| FOV (mm×mm) \| 200×200 \| 220×220 \| 360×280 \| 200×200 \| 203×203 \| 300×270 \| 200×200 \| 400×400 \| 360×360 \| \| No. of signals acquired \| 2 \| 2 \| 1 \| 2 \| 2 \| 1 \| 2 \| 2 \| 1 \| \| b factor (s/mm^2^) \|  \| 50, 1000, 1500 \|  \|  \| 0, 800, 1500 \|  \|  \| 0, 800, 2000 \|  \| |
| TR: Repetition time, TE: Time echo, FOV: Field of view, T2WI: T2-Weighted Imaging, DWI: diffusion weighted imaging, DCE: dynamic contrast-enhanced |

| Table S2 The cut-off for continuous variables in ROC curve analysis |
| --- |
| \| Variables \| cut-off \| AUC (95% CI) \| Sensitivity \| Specificity \| Accuracy \| \| --- \| --- \| --- \| --- \| --- \| --- \| \| SD (mm) \| 8.95 \| 0.688 (0.636-0.740) \| 0.777 \| 0.489 \| 0.608 \| \| CCL/LD \| 1.34 \| 0.723 (0.673-0.772) \| 0.645 \| 0.715 \| 0.686 \| \| LV (ml) \| 1.89 \| 0.706 (0.655-0.757) \| 0.633 \| 0.681 \| 0.661 \| \| NCI_EPE \| 1.5 \| 0.750 (0.704-0.795) \| 0.699 \| 0.664 \| 0.678 \| \| ISUP \| 3.5 \| 0.756 (0.709-0.803) \| 0.675 \| 0.783 \| 0.738 \| \| PI-RADS \| 4.5 \| 0.676 (0.631-0.721) \| 0.735 \| 0.562 \| 0.633 \| |
| ROC: receiver operating characteristic, SD: shortest diameter, CCL: Curvilinear contact length, LD: longest diameter, LV: Lesion volume, NCI_EPE: National Cancer Institute extraprostatic extension, ISUP: International Society of Urological Pathology, PI-RADS: Prostate Imaging Report and Data System version, AUC: area under the curve, CI: Confidence interval |

| Table S3 The comparison of the pT3 and pT2 after scoring by the scoring system in the cases of NCI_EPE grade 2 |
| --- |
| \| Pathological outcome \| \| upgraded scores by the scoring system \| non-upgraded scores by the scoring system \| P value \| \| --- \| --- \| --- \| --- \| --- \| \|  \| N \| 181 \| 85 \| < 0.001 \| \| pT3 \| 135 \| 115 \| 20 \|  \| \| pT2 \| 131 \| 66 \| 65 \|  \| |
| NCI_EPE: National Cancer Institute extraprostatic extension |
